# Preceding Postural Control in Forelimb Reaching Movements in Cats

**DOI:** 10.3389/fnsys.2021.792665

**Published:** 2022-01-18

**Authors:** Mirai Takahashi, Toshi Nakajima, Kaoru Takakusaki

**Affiliations:** ^1^Department of Physiology, Division of Neuroscience, Asahikawa Medical University, Asahikawa, Japan; ^2^Department of Integrative Neuroscience, Faculty of Medicine, The University of Toyama, Toyama, Japan

**Keywords:** postural control, higher order brain function, forelimb reaching, anticipatory postural adjustments, optimization

## Abstract

Postural control precedes the goal-directed movement to maintain body equilibrium during the action. Because the environment continuously changes due to one’s activity, postural control requires a higher-order brain function that predicts the interaction between the body and the environment. Here, we tried to elucidate to what extent such a preceding postural control (PPC) predictively offered a posture that ensured the entire process of the goal-directed movement before starting the action. For this purpose, we employed three cats, which we trained to maintain a four-leg standing posture on force transducers to reach the target by either forelimb. Each cat performed the task under nine target locations in front with different directions and distances. As an index of posture, we employed the center of pressure (CVP) and examined CVP positions when the cat started postural alteration, began to lift its paw, and reached the target. After gazing at the target, each cat started PPC where postural alteration was accompanied by a 20–35 mm CVP shift to the opposite side of the forelimb to be lifted. Then, the cat lifted its paw at the predicted CVP position and reached the forelimb to the target with a CVP shift of only several mm. Moreover, each cat had an optimal target location where the relationship between the cat and target minimized the difference in the CVP positions between the predicted and the final. In this condition, more than 80% of the predicted CVP positions matched the final CVP positions, and the time requiring the reaching movement was the shortest. By contrast, the forelimb reaching movement required a greater CVP shift and longer time when the target was far from the cat. In addition, the time during forelimb reaching showed a negative correlation with the speed of the CVP shift during the PPC. These results suggest that the visuospatial information, such as the body-environment interaction, contributes to the motor programming of the PPC. We conclude that the PPC ensures postural stability throughout the action to optimize the subsequent goal-directed movements. Impairments in these processes may disturb postural stability during movements, resulting in falling.

## Introduction

Posture means the static position of any part of the body, and movements are the transition from one posture to another (Brooks, 1986). To achieve the goal-directed movement, there is a need to prepare an appropriate posture before the onset of the purposeful action and to maintain body equilibrium during ongoing movement so that the subject stabilizes the body’s center of mass until the end of the task (Horak, 2006). Because movements accompany continuous changes in one’s body and environment, a series of the postural control requires higher-order brain function in order to predict these changes. Accordingly, dysfunction of the higher-order brain functions due to aging and neural disorders is suggested to elicit gait failure and falling because of impairment of these postural control processes (Snijders et al., 2007). Then, the critical question is how such a “predictive and preparatory posture control” is accomplished. Anticipatory postural adjustment (APA) is known as an example of posture control that precedes purposeful action. Indeed, plenty of studies have been shown that the APA is disturbed when various neural structures, such as the cerebellum (Timmann and Horak, 2001; Bruttini et al., 2015), basal ganglia (Jacobs et al., 2009b; Ng et al., 2013), and cerebral cortex and their adjacent areas are damaged (Delafontaine et al., 2019). However, it has not been well understood how APA guarantees the postural equilibrium during and at the end of the subsequent goal-directed movement. Moreover, there was no substantial evidence of how the above brain structures regulate the APA. Accordingly, we still cannot describe the relationship between the disturbances in APA and damages in any brain structures resulting in falling.

It has been shown that APA is triggered not only by whole body movement such as step initiation (McIlroy and Maki, 1999; Delafontaine et al., 2019), but also by focal body movements such as upper limb rising (Belen’kii et al., 1967) and handle pulling (Cordo and Nashner, 1982). It is also observed in quadrupeds, such as rodents (Yamaura et al., 2013) and cats (Schepens and Drew, 2003). Regardless of whether human beings or animals, the APA is often expressed by postural reaction that resists changes in the center of gravity accompanying the movements. Using kinemato-dynamic measurement procedures, Hugon et al. (1982) and Varghese et al. (2016) temporally separated APA, which was characterized by the inverse postural reaction, from purposeful action of the entire movement. Accordingly, most studies have focused on the mechanisms of generating such an inversive reaction as a parameter of the APA. However, there was a report showing that younger healthy subjects without the inversive reaction maintained postural equilibrium well in response to the gait initiation (Hyodo et al., 2012), indicating that the inversive postural reaction is not necessary for APA. Therefore, neuronal mechanisms that generate “preceding postural control (PPC),” other than the inversive reaction may exist so that stable posture is ensured from the onset to the end of movements. We hypothesize that the mechanisms require satisfying the following two conditions. The first condition is that the posture offered by the PPC before starting the goal-directed movement ensures postural stability during and until the end of the movement. Namely, there is a need to predict and provide appropriate postural control for the whole period of the action before its starting. The second is that the PPC alters depending on the relationship between the subject and one’s surrounding circumstances.

The present study was designed to verify the above hypothesis using cat experiments. For this purpose, we trained three cats to perform the forelimb reaching movement task from a four-leg standing posture. Then, we evaluated postural changes in the whole movement by investigating the changes in spatiotemporal parameters of the center of vertical pressure (CVP), an index of the center of gravity. Here, we tried to elucidate the following three issues. The first issue was to examine to what extent the PPC predicted the CVP position at the end of the reaching movement. The second was to investigate whether and how the PPC was altered depending on the spatial relationship between the target and the cat. It was also essential to examine whether each cat had an optimal target position where the cat achieved the task with high performance. Third, we tried to identify which spatiotemporal parameters of the PPC related to the degree of the task performance.

We demonstrated the following characteristics of the PPC in the forelimb reaching task in the cat. First, the PPC largely achieved postural alteration of the entire movement. Specifically, while the CVP shift during the PPC was more than 20 mm, it was several millimeters during the forelimb reaching. Second, not only were CVP positions altered by changes in the target position, but there was also an optimal target-subject condition in each cat where the difference in the CVP positions between the predicted and the final was the minimum. In this condition, more than 80% of the expected CVP positions matched the final CVP positions, and the time during the forelimb reaching was the shortest. Third, the time during reaching showed a negative correlation with the speed of the CVP shift during the PPC. These results suggest that motor programs based on cognitive visuomotor processing achieve both voluntary movement and the PPC. The PPC, therefore, may ensure a stable posture of the subsequent purposeful movement and affect its performance. The spatiotemporal parameters of PPC can be an index of postural stability and also, a fall-risk marker for predicting falls due to higher-order brain dysfunction. In a series of studies, we will elucidate in animal experiments how damage to various brain structures modifies PPC. This study is the first and fundamental study for that purpose.

## Materials and Methods

### Animals

The study was based on three adult female cats, which weighed from 2.2 to 2.4 kg. These cats were obtained from a laboratory animal supplier and bred at the animal facility of Asahikawa Medical University, where they were kept in individual cages under constant temperature and light-dark cycles. All the procedures of the present experiments were approved by the Animal Studies Committee of Asahikawa Medical University (approval number; R3-116) and were following the Guide for the Care and Use of Laboratory Animals (NIH Guide), revised 1996.

### Experimental Setup

**FIGURE 1:**
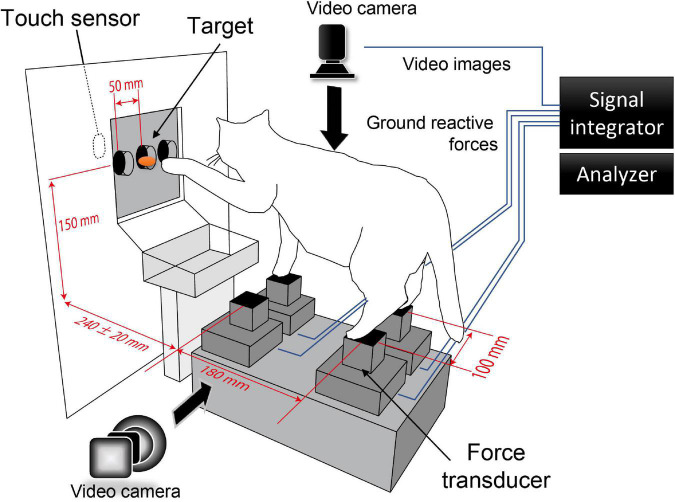
Experimental setup. The cat maintained a standing posture in the platform and performed forelimb reaching movement to the target in front. The task was performed under the nine different target conditions (distance: 240 ± 20 mm, direction: center, left 50 mm, right 50 mm). The ground reactive force and the video from dorsal and lateral views were integrated and recorded by the signal integrator. The moment of target reaching was detected by the touch sensor installed behind the target.

The cat maintained a standing posture in the platform and performed forelimb reaching movements to the target in front (Figure 1). The ground reactive force exerting in each foot was measured by force transducers (TEAC, with sampling rate of 10 kHz) placed beneath each foot. The length of a side of each transducer was 40 mm in which size was just enough to place each foot. Four transducers were placed on a horizontal plane so that the centers of them formed a rectangle with its antero-posterior and left-right edges spanning 180 mm and 100 mm, respectively. This setup allowed the cat naturally maintain its standing posture. A food pellet, which is the target, was put into a cylinder (30 mm diameter) fixed to the board in front, and the cat reached the target by either forelimb. In a standard condition, the target location was 240 mm in the distance from the forelimb force transducers with a height of 150 mm, mainly equal to the cats’ shoulders. In addition, we investigated how the cat’s postural control altered during the forelimb reaching task in response to changes in the target’s spatial position, which we moved in the left-right and anterior-posterior directions with 50 and 20 mm, respectively. We detected the moment the forelimbs reached the touch sensor, which was fixed to the back of the board and attached to the feeding cylinder.

### Recordings, Measurements, and Parameters

We trained the cats three times a week for 3 months or more until the cat steadily performed the reaching task. After that, the experiment was carried out three times a week. A fasting period of 0.5–1 day was set the day before the experiment, paying attention to the weight fluctuation and health of the cat. Each experimental session continued as long as the cat tackled the task. Changes in ground reactive force of all trials of the forelimb reaching task were recorded in LabChart software by PowerLab system (AD Instruments) and stored in the hard disk for later off-line analyses. In addition, we simultaneously recorded the dorsal and lateral views of the cat’s movements by the Powerlab system at a rate of 30 frames/s (Figure 1).

**FIGURE 2:**
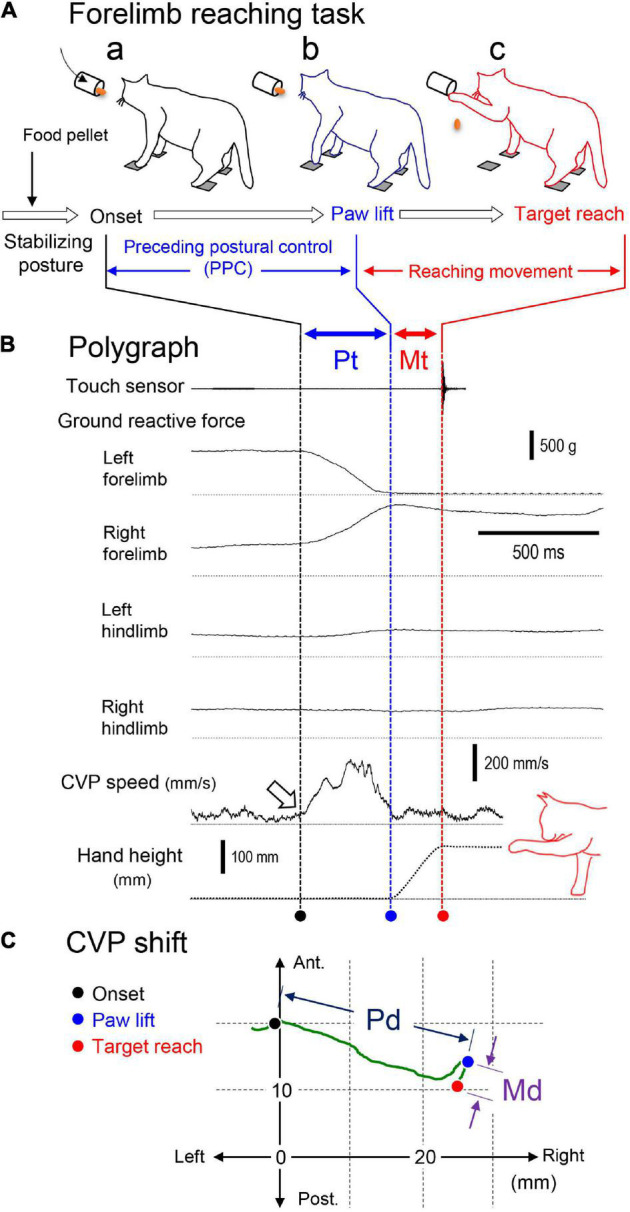
Recordings, measurements, and parameters of the forelimb reaching task of the cat. **(A)** Illustrated cat figures of the forelimb reaching task at the onset of the preceding postural control **(a)**, paw lift **(b)**, and target reach **(c)**. The task was composed of three phases as “stabilizing posture,” “preceding postural control (PPC),” and “reaching movement.” **(B)** Polygraphic recordings of the task. Vertical lines and circles below the with different colors indicate the moment of onset (black), paw lift (blue), and target reach (red). The periods of time from the onset to paw lift and from the paw lift to target reach were determined as postural time (Pt) and movement time (Md), respectively. **(C)** The shift of the center of vertical pressure (CVP) in the above trial. Circles with different colors indicate positions of the CVP at onset (black), paw lift (blue), and target reach (red). The distance of the CVP between the onset and paw lift was determined as postural distance (Pd), and that between the paw lift and target reach was determined as movement distance (Md).

Figure 2 shows an example of polygraphic recordings of a forelimb reaching movement, which was composed of three phases as “stabilizing posture,” “PPC,” and “reaching movement.” In the first stabilizing posture phase, the cat kept stabilizing posture by attending to the food pellet as a target put into the cylinder. The second PPC phase corresponded from the onset of postural change (a dashed line with a filled circle) to either forelimb’s paw lift (Figure 2Aa). When the ground reactive force became zero, we determined this moment as paw lift (a dashed line with a blue circle). Finally, we defined the period from the paw lift to the target reach as the reaching movement phase. The touch sensor signal (indicated by a dashed line with a red circle) indicated the end of the task. Then, the cat dropped the food pellet on the tray in front to get the reward. The cat, after that, again took stabilizing posture so that it started the subsequent trials. Thus, we changed target conditions every 30 trials. We further determined the period that required the PPC as “preceding postural control time (Pt)” and that to the forelimb reaching movement as “movement time (Mt).” The center of vertical pressure (CVP), a parameter of the center of gravity, was calculated by the following equation from the ground reactive forces generated by each of four force transducers. We used changes in CVP positions associating with the forelimb reaching task as the index of posture change.


$$\text{CVP}=\left({\text{CVP}}_{L\,R},{\text{CVP}}_{A\,P}\right)$$

$$C\,V\,P_{L\,R}=\frac{F_{r\,F\,L}+F_{r\,H\,L}-F_{l\,F\,L}-F_{l\,H\,L}\,\left[\text{kg}\cdot m/s^{2}\right]}{M_{c\,a\,t}\,g\,\left[\text{kg}\cdot m/s^{2}\right]}\times 50\,\left[\text{mm}\right]$$

$$C\,V\,P_{A\,P}=\frac{F_{l\,F\,L}+F_{r\,F\,L}-F_{l\,H\,L}-F_{r\,H\,L}\,\left[\text{kg}\cdot m/s^{2}\right]}{M_{c\,a\,t}\,g\,\left[\text{kg}\cdot m/s^{2}\right]}\times 90\,\left[\text{mm}\right]$$

The distance between the centers of the force transducers were 100 mm for the left-right direction, and 180 mm for the antero-posterior direction. Therefore, the coordinates of the CVP positions were calculated by multiplying the distributions of the ground reaction force in the left-right and antero-posterior direction by 50 and 90 mm, respectively. *CVP*_*LR*_, CVP at the left-right direction on the horizontal axis; *CVP*_*AP*_, CVP at the anterior-posterior direction on the vertical axis; *F*_*rFL*_, ground reactive force at the right forelimb; *F*_*rHL*_, ground reactive force at the right hindlimb; *F*_*lFL*_, ground reactive force at the left forelimb; *F*_*lHL*_, ground reactive force at the left hindlimb; M*_*cat*_*, body weight of the cat.

To characterize the postural control of the forelimb reaching task, we selected positions of CVP at the moments of the “onset” of postural changes, namely, “paw-lift” and “target-reach” (Figure 2C). Then, we calculated the distance between these CVP positions according to the coordinates. Moreover, we defined the difference in CVP positions between “onset (a filled circle)” and “paw-lift (a blue circle)” as “a CVP distance for preceding postural control (Pd),” and that between “paw-lift (a blue circle)” and “target-reach (a red circle)” as “a CVP distance for postural control associating with reaching movement (Md).” Similarly, we defined the periods requiring the preceding postural control and the forelimb reaching movement as preceding postural control time (Pt) and movement time (Mt), respectively. We also defined the mean speed of CVP shift during the PPC and forelimb movement as postural CVP speed (P-CVPs) and movement CVP speed (M-CVPs), respectively. The P-CVPs and M-CVPs were calculated by Pd/Pt and Md/Mt, respectively. As indicated by an open arrow in Figure 2B, the rapid increase in the CVP speed corresponded to the onset of the PPC.

### Statistics

We employed R [R Core Team (2018). R: A language and environment for statistical computing. R Foundation for Statistical Computing, Vienna, Austria]^1^ for all statistical analyses. The mean value is expressed by mean ± standard deviation (SD). We judged the case where the *p*-value was less than 0.05 as significant. When conditions were with different target locations, we used the distance of the targets in the anterior-posterior direction (240 mm ± 20 mm) and left-right direction (50 mm left or right) as explanatory variables to examine the statistical significance of each parameter. Student’s *t*-test was used to compare the changes in each parameter under two specific conditions. In addition, Pearson’s correlation coefficient was calculated to investigate the relationship between the parameters under the nine target conditions. Those whose absolute value of the correlation coefficient was more than 0.4 and were significant were judged to be correlated (Evans, 1996). We calculated the 95% confidence interval of the CVP distributions at each moment (Onset, Paw lift, and Target reach) and displayed it as a confidence ellipse.

## Results

### Preceding Postural Control Began With a Decrease in the Load on the Forelimb to Be Lifted

**FIGURE 3:**
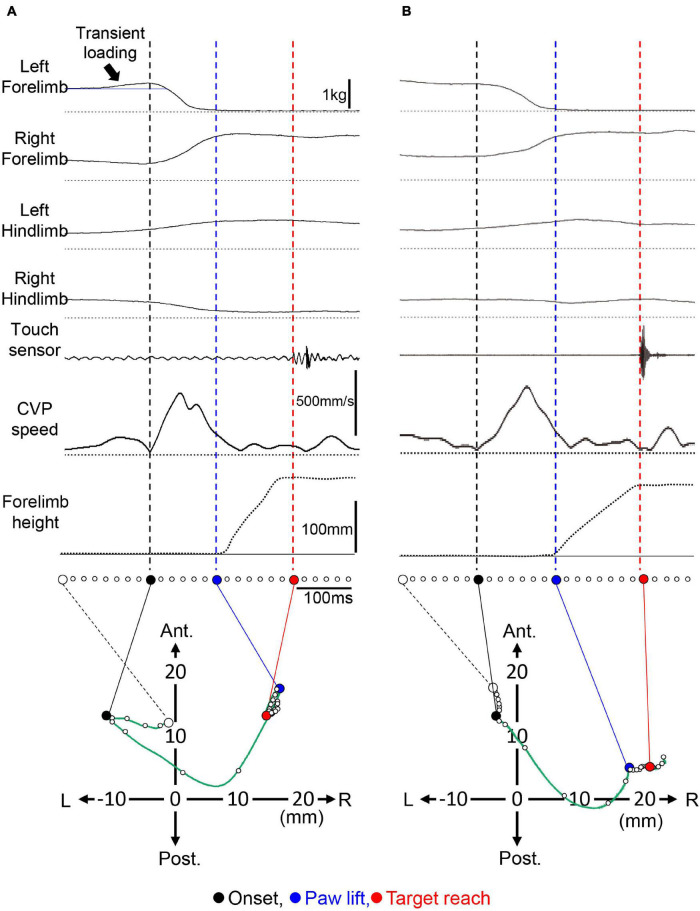
Forelimb reaching movement with and without transient loading of the forelimb before PPC. **(A)** An example of the trial with a transient. An arrow indicates the transient loading. The presence of the loading was reflected by the CVP shift before the onset of the PPC from an open circle to a filled circle. An open circle in the CVP shift indicates the CVP position at the stabilizing posture of the cat before the onset of the transient loading. **(B)** A representative trial without the transient loading. The format is the same as panel **(A)**. L, left; R, right; Ant, anterior; Post, posterior.

As described in introduction, most studies so far focused on the inversive kinetic reaction, which is characterized by the transient load increase acting on the forelimb to be lifted, as is the APA. However, through the period of this study, we have encountered many trials without such a transient loading. One example of the forelimb reaching task in the same cat are shown in Figure 3. The trial in Figure 3A showed a transient increase in the ground reactive force exerting the left forelimb (indicated by an arrow), which preceded the onset of the PPC. The temporary load increase reflected the CVP shift from the first position (an open circle) to the left with a distance of approximately 10 mm. However, a trial in Figure 3B did not exhibit such an apparent transient loading. Whether the transient loading increase existed or not, the PPC, characterized by unloading the left forelimb and loading the right forelimb, started. The times required to the PPC (Pt) were 125 and 150 ms for trials (A) and (B), respectively. The changes in the loading of the forelimbs and those of the hindlimbs evoked a rapid increase in the shift of CVP speed during the PPC. For trial (A), the maximum speed of the CVP shift was 475 mm/s with a mean CVP speed (P-CVPs) of 217 mm/s. These were 503 and 156 mm/s for (B). During this period, CVP moved the position from a filled circle to a blue circle. For (A), the CVP moved to the right and anterior directions with 27 and 3 mm, respectively. For (B), it moved to 22 mm right and 8 mm posterior. Therefore, a CVP shift during PPC, which corresponded to the Pd, was calculated as 27.2 mm for (A) and 23.4 mm for (B). Then, the PPC was followed by forelimb movements. The Mt and Md were 140 ms and 5.2 mm, respectively, for (A). They were 155 ms and 2.8 mm for (B). The mean CVP movement speed (M-CVPs) was 37 mm/s for (A) and 18 mm/s for (B). Accordingly, the Md and M-CVPs were much smaller than Pd and P-CVPs, respectively. While we examined more than 2,500 trials in three cats, the trials that accompanied the transient loading before the PPC was less than 60%. It was observed to be 56.9% (338/594 trials) for Cat 1, 57.1% (432/756 trials) for Cat 2, and 26.8% (316/1227 trials) for Cat 3. These findings indicate that the presence of the inversive postural reaction is not essential for PPC. Therefore, we defined that the PPC began with a reduction of the load on the forelimb to be lifted regardless of the presence or absence of the inversive reaction. Then, we performed the subsequent analysis according to this definition.

### Parameters of Center of Vertical Pressure During Forelimb Reaching Task

**FIGURE 4:**
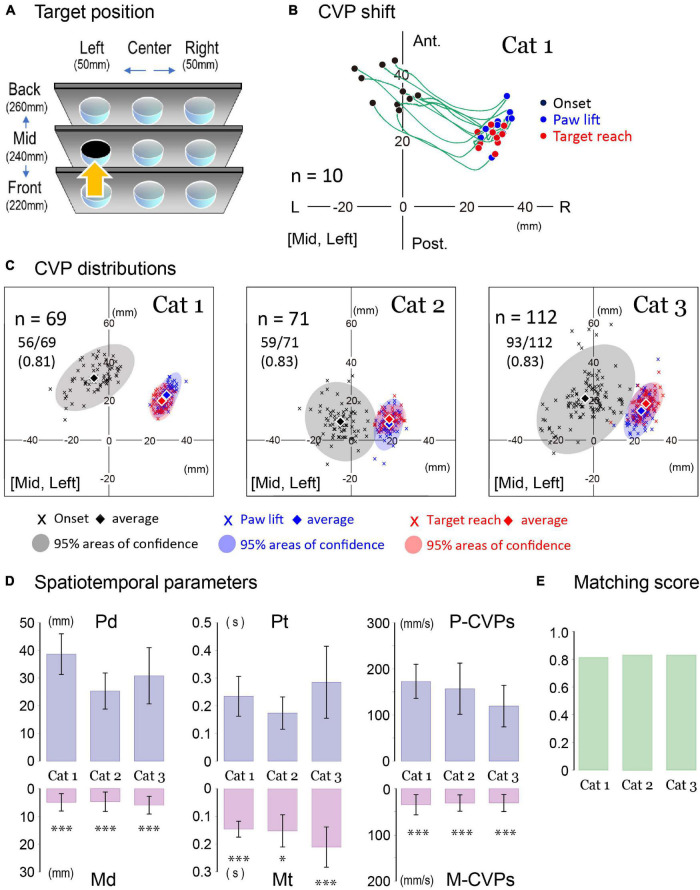
Parameters of forelimb reaching task. **(A)** An arrow indicates a target location. **(B)** CVP shifts of ten consecutive trials of the left forelimb reaching movement in Cat 1. The CVP positions at the onset of PPC, paw lift, and target reach were indicated by black, blue, and red circles. The green lines are the trajectory of the CVP shift in each trial. **(C)** The distributions of CVP at each moment in three cats. Cross marks indicate CVP positions in each moment with different colors (onset: black, paw lift: blue, target reach: red). Translucent colored ellipses represent 95% areas of confidence. Diamonds indicate the average position of the CVP at each moment. **(D)** From left to right, comparisons were made between Pd and Md (left), Pt and Mt (center), and postural CVP speed (P-CVPs) and movement CVP speed (M-CVPs). These data were obtained from 69 trials in Cat 1, 71 trials in Cat 2, and 112 trials in Cat 3. The Pd was 38.6 ± 7.4 mm for Cat 1, 25.3 ± 6.5 mm for Cat 2, and 30.8 ± 10.2 mm for Cat 3. The Md was 4.9 ± 3.1 mm for Cat 1, 4.7 ± 3.5 mm for Cat 2, 5.9 ± 3.2 mm for Cat 3. The Pt was 0.234 ± 0.072 s for Cat 1, 0.173 ± 0.058 s for Cat 2, and 0.285 ± 0.130 s for Cat 3. The Mt was 0.146 ± 0.029 s for Cat 1, 0.152 ± 0.058 s for Cat 2, and 0.210 ± 0.073 s for Cat 3. P-CVPs and M-CVPs were 173 ± 37 mm/s and 34.5 ± 21.9 mm/s for Cat 1, 157 ± 55 mm/s and 30.9 ± 17.7 mm/s for Cat 2, 119 ± 45 and 30.7 ± 18.4 mm/s for Cat 3, respectively. In each cat, there was a significant difference between Pd and Md, Pt and Mt, and P-CVPs and M-CVPs for each cat. The bar plots show each spatiotemporal parameter in each cat. Box and bar indicate the Mean ± SD. Asterisk(s) indicate a significant difference. *p < 0.05, ***p < 0.001. **(E)** Matching score of CVP at paw lift and CVP at target reach in three cats. L, left; R, right; Ant, anterior; Post, posterior.

Next, we investigated whether the changes in spatiotemporal parameters associated with CVP shifts were common to all animals. Examples are shown in Figure 4 where each cat achieved the forelimb reaching movement to the target at the mid-left location (an arrow in Figure 4A). Traces in Figure 4B showed CVP shift in 10 consecutive trials of the forelimb reaching task of Cat 1. Before starting PPC, each CVP (CVP_onset_; filled circles) was distributed between 20 and 40 mm in the anterior-posterior coordinate and −20 and 8 mm in the left-right coordinate. When starting the PPC, CVP shifted 25–35 mm in the right-posterior direction to the position where paw lift would occur (CVP_lift_; blue circles). Thus, the positions of the CVP_lift_ in 10 trials existed within the limited area surrounded by the coordinates of the 22–35 mm right and 15–33 mm anterior. Then, the cat started lifting the paw to extend the forelimb so that the CVP shifted to the position where the cat reached the target (CVP_reach_; red circles). Despite dynamic limb movements in this process, CVP in any trial only moved within a few millimeters.

Using this procedure, we investigated the changes in CVP positions of the forelimb reaching the same target location in the three cats. Each graph in Figure 4C shows CVP_onset_ (black cross mark), CVP_lift_ (blue cross mark), and CVP_*reach*_ (red cross mark) together with translucent colored ellipses that indicate 95% areas of confidence for Cat 1 (left), Cat 2 (center), and Cat 3 (right). In each, these diamond marks with different colors indicate average positions of the CVP of all trials at each moment of interest. While the average positions of the CVP_onset_ (diamonds) in each cat primarily existed at 5–10 mm to the left, those were different in the anterior-posterior direction. They were approximately 32, 9, and 21 mm rostral to the reference point for the Cats 1, 2, and 3, respectively. Moreover, the size of the 95% areas of confidence for CVP_onset_ (light gray) differed among the animals. It was approximately 940 mm^2^ for Cat 1, 1,200 mm^2^ for Cat 2, and 2,100 mm^2^ for Cat 3. Compared to the CVP_onset_, the size of the areas consisting of the CVP_lift_ and CVP_reach_ were much smaller. For Cat 1, 2, and 3, the CVP_lift_ areas (faded blue) were approximately 210, 310, and 360 mm^2^ and the CVP_reach_ areas (faded red) were about 190, 200, and 290 mm^2^ in size, respectively. We further observed that the two areas considerably overlapped in each cat (Figure 4C). For example, when we counted the number of CVP_lift_ (blue cross mark) within areas of 95% confidence of CVP_*reach*_ (faded red), it was found to be 56 out of 69 (81%) for Cat 1, 59 out of 71 (83%) for Cat 2, and 93 out of 112 (83%) for Cat 3. We defined these percentages as the matching score of the CVP_lift_ and CVP_reach_. That is, as shown in Figure 4E, they were 0.81, 0.83, and 0.83 in Cats 1, 2, and 3, respectively.

In Figure 4D, we compared spatiotemporal parameters of CVP shift in each cat during the PPC and during forelimb reaching in the upper and lower graphs, respectively. The mean distance of CVP shift in each cat was more than 25 mm during the PPC (Pd). However, it was less than 6 mm during forelimb movement (Md) (left in Figure 4D), indicating that postural change during forelimb reaching was more petite than during PPC. Concerning times requiring for the PPC (Pt) and forelimb movement (Mt), the former was longer than the latter in each cat (center in Figure 4D). Moreover, CVP speed during PPC (P-CVPs) was much faster than that during forelimb movement (M-CVPs) in each animal (right in Figure 4D). These common findings in the three cats suggest that the cat did not evoke significant postural change during the goal-directed movement, but had achieved appropriate postural alteration during PPC even before forelimb movement. In addition, because the matching score was more than 0.8 (Figure 4E), we postulate that the cat had already predicted to determine posture at the target reaching (CVP_reach_) until lifting its forelimb (CVP_lift_) with a much higher probability.

### Changes in Spatiotemporal Parameters of the Center of Vertical Pressure Induced by Different Target Conditions

**FIGURE 5:**
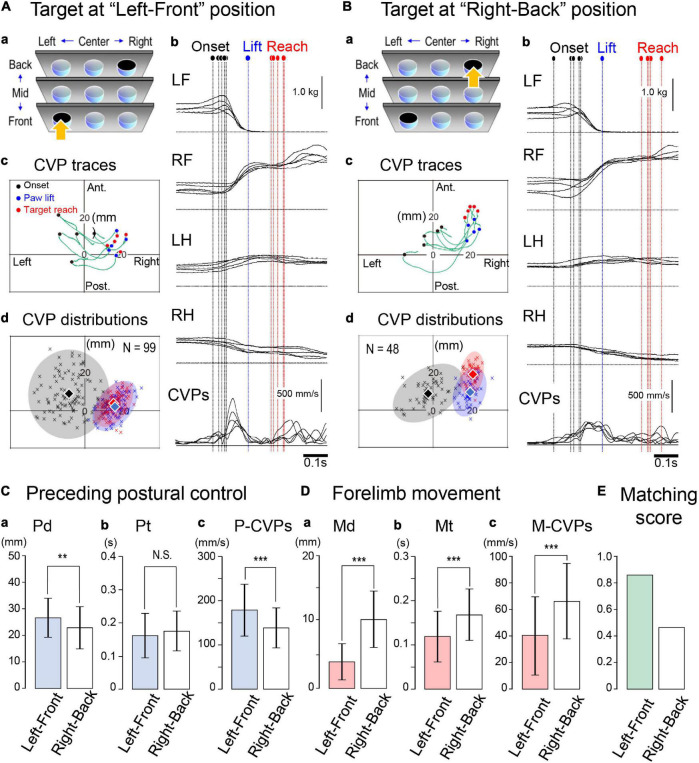
Changes in spatiotemporal parameters of the CVP induced by different target conditions. **(A)** Forelimb reaching the target at the “left-front” location. **(a)** The arrow indicates the target location. **(b)** Polygraphic recordings of consecutive five trials, which were aligned at the paw lift. Colored dashed lines indicate the moments of onset (black), paw lift (blue), and target reach (red). **(c)** Traces of CVP shifts in the five trials. Each circle indicates the CVP position at each moment. **(d)** The CVP distribution of 99 trials in this target condition. Translucent colored ellipses represent 95% areas of confidence. The diamonds indicate the average position of the CVP at each moment. **(B)** Forelimb reaching the target at the “right-back” location. From panels **(a–d)**, data are presented in the same format as panel **(A)**. **(C)** Comparison of parameters relating to the PPC between the two conditions. Panels **(a–c)** are Pd, Pt, P-CVPs, respectively. **(D)** Comparison of parameters relating to the forelimb movement between the two conditions. Panels **(a–c)** are Md, Mt, and M-CVPs, respectively. **(E)** Comparison of matching scores between the two conditions. Box and bar indicate the Mean ± SD. Asterisk(s) indicate a significant difference. **p < 0.01, ***p < 0.001.

Next, we examined whether and how the difference in the target location altered the entire process of the forelimb reaching. Then, each cat performed the forelimb reaching movement to the nine targets placed at different locations in the rostrocaudal and left-right directions. Representative findings are shown in Figures 5A,B, where Cat 2 reached the target at “left-front” and “right-back” locations, respectively. Against the left-front target condition, the cat started the task with a PPC time (Pt) of less than 0.15 s and a CVP speed of 300–600 mm/s. Subsequently, the cat reached the target with a movement time (Mt) between 0.09 and 0.13 s (Figure 5Ab). Concerning the CVP position (Figure 5Ac), the CVP_onset_ of each trial (filled circles) was broadly distributed. During the PPC, they moved a distance of 20–30 mm in right or right-posterior directions to gather to the area of 15–25 mm right and ∼10 mm anterior to the coordinate to arrive at the CVP_lift_ positions (blue circles). Subsequently, each CVP moved approximately 3 mm, arriving at the positions of the CVP_reach_ (red circles). The distribution of the CVP_onset_, CVP_lift_, and CVP_reach_ of 99 trials in this condition are illustrated in Figure 5Ad. The average position of the CVP_onset_ was 8.6 mm left and 8.7 mm anterior to the coordinate (filled diamond). A gray colored circle corresponded to the area of 95% confidence with a radius of 20 mm centered on the point representing the average CVP_onset_ position. On the other hand, areas representing the distribution of the CVP_lift_ and CVP_reach_ were smaller than the area of the CVP_onset_. The average position of the CVP_lift_ was 16.2 mm right with 2.6 mm anterior, and that of the CVP_reach_ was 15.5 mm right with 5.0 mm anterior. Because of their close positions, there was considerable overlap, or matching, between these two areas.

In the “right-back” target condition (Figure 5Ba), the cat required approximately 0.2 s for the PPC and 0.2 s for the subsequent forelimb movement (Figure 5Bb). The CVP speed during the PPC (P-CVPs) was less than 300 mm/s. Along with the PPC progress, the CVP of the five trials first moved to the right and then turned to the anterior direction so that the CVP arrived at the CVP_lift_ position (Figure 5Bc). The forelimb movement further took the CVP position 5–10 mm forward to achieve the movement, resulting in the separation of the CVP_lift_ and CVP_reach_ positions. Figure 5Bd shows distributions of each CVP position of 48 trials. The average positions were 2.8 mm left and 9.2 mm anterior for the CVP_onset_, 19.6 mm right and 9.7 mm anterior for the CVP_lift_, and 20.9 mm right and 19.7 mm anterior for the CVP_reach_. Consequently, the average CVP_onset_ was approximately 6 mm to the right in the right-back condition compared to the left-front condition. Moreover, the average CVP_lift_ and CVP_reach_ positions in the latter condition were approximately 7 and 15 mm anterior to the former condition, respectively. Thus, in the latter condition, the distributions of CVP_lift_ (faded blue) and CVP_reach_ (faded red) had a slight overlap, or were less matching, which reflected the considerable difference of 10 mm between the average CVP positions at the lift (blue diamond) and reach (red diamond).

We compared parameters between the above two conditions during the PPC (Figure 5C) and forelimb movements (Figure 5D). The distance of the CVP shift during PPC (Pd) was longer (*p* < 0.01, *t*-test) in the left-front condition (26.6 ± 7.3 mm, *n* = 99) than in the right-back condition (22.8 ± 7.8 mm, *n* = 48, Figure 5Ca). On the other hand, there was no difference in the time required for PPC (Pt) between the two conditions (0.161 ± 0.068 s, *n* = 99 for left-front; 0.175 ± 0.062 s, *n* = 48 for right-back, Figure 5Cb). The speed in the CVP during the PPC (P-CVPs) was, accordingly, faster (*p* < 0.001, *t*-test) in the left-front condition (179 ± 59 mm/s, *n* = 99) than right-back condition (138 ± 45 mm/s, *n* = 48; Figure 5Cc). During forelimb movement, the former condition showed a shorter (*p* < 0.001, *t*-test) distance of the CVP shift (Md, 4.1 ± 2.8 mm, *n* = 99, Figure 5Da), a shorter (*p* < 0.001, *t*-test) period of the time (Mt, 0.118 ± 0.058s, *n* = 99, Figure 5Db), and faster (*p* < 0.001, *t*-test) speed in the CVP shift (M-CVPs, 41 ± 29 mm/s, *n* = 99, Figure 5Dc) than the latter condition (10.4 ± 4.3 mm, *n* = 48 for Md, 0.168 ± 058 s, for Mt, and 66 ± 29 mm/s, *n* = 48 for M-CVPs). The matching score, which was the number of CVP_lift_ relative to the number of CVP_reach_ in the red colored area, was 86% in the former condition. However, it was only 46% in the latter. Therefore, the difference in the target location altered spatiotemporal parameters relating to both the PPC and forelimb movement.

Presentation of the targets with different locations considerably altered forelimb reaching task parameters in Cat 2. Then, we tried to answer whether targets at different positions modified the parameters of the other two animals. It was further critical to elucidate what parameter changes were common to all three animals.

### Changes in the Distribution of Center of Vertical Pressure Induced by Different Target Conditions

**FIGURE 6:**
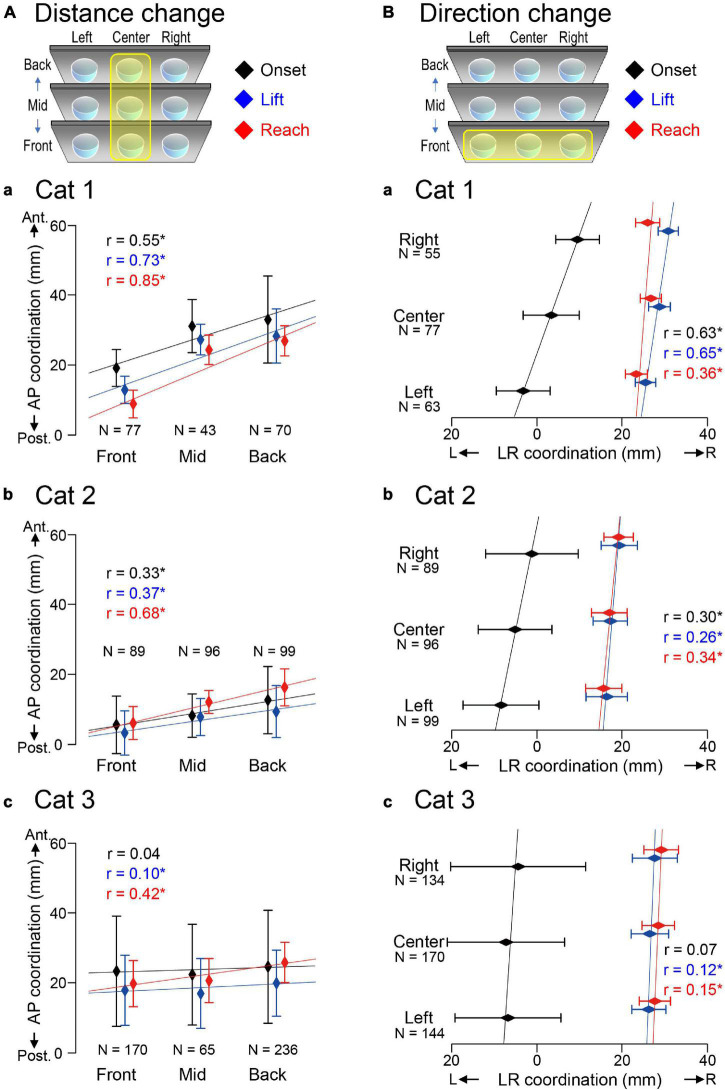
Alterations of CVP induced by different target conditions. **(A)** Alterations in CVP on the anterior-posterior coordination depending on the different target distances. Each rhombus and bar indicate the Mean + SD of CVP, at the moment of onset (black), paw lift (blue) and target reach (red). Each regression line represents the target condition as the explanatory variable and CVP as the objective variable. “*r*” indicates the correlation coefficient. **(B)** Alterations in CVP on the Left-Right coordination depending on the different target directions. The format is the same as panel **(A)**. Asterisk indicate a statistical significance of *p* < 0.05.

Figure 6 and Table 1 showed that different target conditions altered CVP positions of interest in all three cats. Specifically, the CVP_lift_ (blue) and CVP_reach_ (red) moved forward in all cats by moving the center target from front to back (Figures 6Aa–c). Similarly, the CVP_lift_ (blue) and CVP_reach_ (red) moved to the right in all cats when positions of the target in the front row moved from left to right (Figures 6Ba–c). In addition, CVP_onset_ (black) also moved forward or to the right in Cats 1 (Figures 6Aa,Ba) and 2 (Figures 6Ab,Bb) in the above two conditions. However, the CVP_onset_ of Cat 3 (Figures 6Ac,Bc) did not move in either target condition.

**TABLE 1:** Correlation coefficients between target conditions and center of vertical pressure (CVP) positions.

		Target conditions (explanatory variable)					
	
		Distance (Front - Mid - Back)			Direction (Left - Center - Right)		
Cat number		Cat1	Cat2	Cat3	Cat1	Cat2	Cat3
**CVP**							
CVP_onset_	L⇔R	0.22*	0.05	0.14*	0.63*	0.30*	0.07
	A⇔P	0.55*	0.33*	0.04	0.05	−0.26*	−0.07
CVP_lift_	L⇔R	0.60*	0.11	0.15*	0.65*	0.26*	0.12*
	A⇔P	0.73*	0.37*	0.10*	0.39*	0.07	0.02
CVP_reach_	L⇔R	0.59*	0.30*	0.31*	0.36*	0.34*	0.15*
	A⇔P	0.85*	0.68*	0.42*	0.15*	0.42*	0.16*
							*p < 0.05
		**Number of trials in each target condition**					
	
		**Front**	**Mid**	**Back**	**Left**	**Center**	**Right**

Cat 1		77	43	70	63	77	55
Cat 2		96	98	75	99	96	89
Cat 3		170	65	236	144	170	134

The above findings suggest that changes in CVP_lift_ and CVP_reach_ in the three cats shifted depending on the target location alterations. The findings also support our hypothesis that CVP_lift_ can be offered in anticipation of CVP_reach_. Moreover, the CVP_onset_ of Cats 1 and 2, but not Cat 3, changed depending on the target condition difference. Therefore, the difference in the target conditions may also affect waiting or stabilizing posture before starting the PPC.

### Comparison of All Parameters That Altered Depending on the Difference in Target Conditions

**FIGURE 7:**
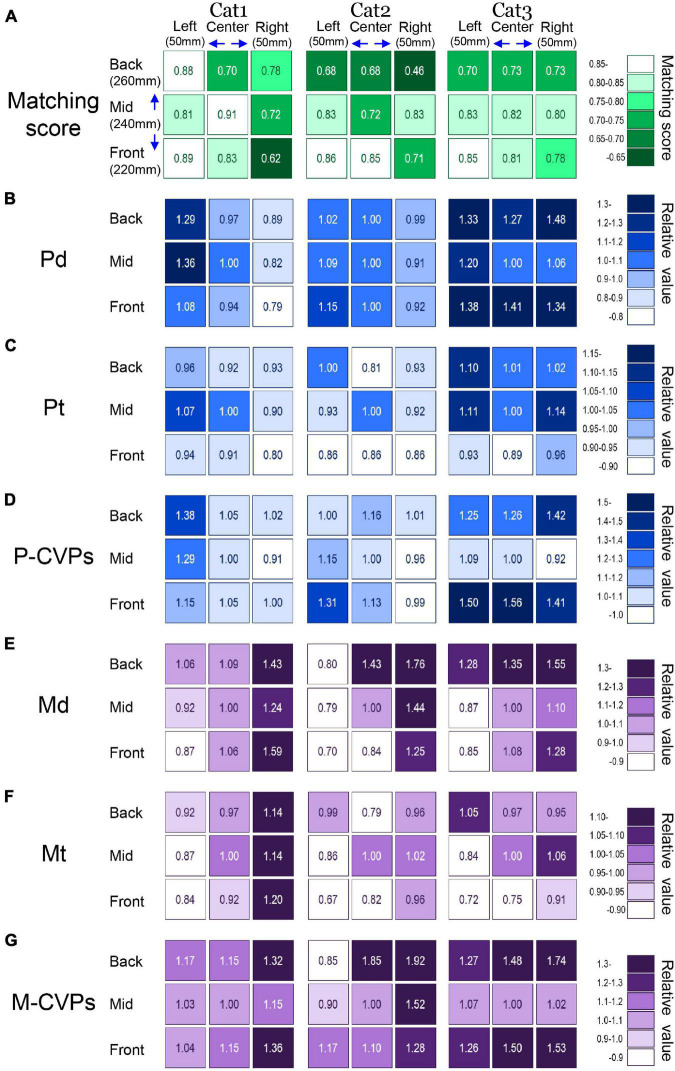
Comparison of all parameters that altered depending on the difference in target conditions. **(A)** Matching scores in each target condition of three cats. Panels **(B–G)** show the relative value of each spatiotemporal parameter when the mean value in the standard target condition (Mid-Center) is 1.0. See text for detailed explanations.

Finally, we elucidated the optimal target location for each cat to achieve the task with the most stable posture. The optimal target location can be determined by investigating to what extent PPC offered the CVP_lift_ that matched the location of the CVP_reach_. It is also critical to identify spatiotemporal parameters contributing to the optimal postural control common to all three cats. To clarify these issues, we visualized the changes in the parameters depending on the different target conditions using shades of colors, as shown in Figure 7.

Figure 7A shows the matching score. This was 0.62–0.91 for Cat 1, 0.46–0.86 for Cat 2, and 0.70–0.85 for Cat 3. When the target was at the left-front, the matching score of all cats was higher than 85%. On the other hand, the target location at the right-back reduced the matching in each cat. In Figures 7B–G, we set 1.0 for the mean value of each parameter under the reference target location at the mid-center. Then, each parameter under other eight target locations was expressed as a relative value against the reference condition. Changes in the target locations considerably altered each parameter. The Pd, which is the distance shift during PPC, changed in the range of 0.79–1.36 for Cat 1, 0.91–1.15 for Cat 2, and 1.00–1.48 for Cat 3 (Figure 7B). In Cat 1, the Pd was relatively small when targets were placed in the right, but it was rather large when the targets were placed in the left. While Pd in Cat 2 exhibited a small change with a relative value of less than 1.15 under any target locations, Cat 3 had a rather large value of more than 1.20 for target locations other than the mid-center and mid-right. Moreover, target locations with the smallest and largest Pd were different in each cat. The Pt, which corresponded to the time requiring PPC, was in the ranges of 0.80–1.07 for cat 1, 0.81–1.00 for cat 2, and 0.89–1.14 for cat 3 (Figure 7C). While targets in the front row exhibited a relative value of less than 0.9 in all cats, target locations with the smallest and largest Pt differed among the three animals. The P-CVPs, a mean CVP speed during PPC, changed in the range of 0.91–1.38 for Cat 1, 0.96–1.31 for Cat 2, and 0.92–1.56 for Cat 3 (Figure 7D). When the target was placed at the mid-right, all three cats exhibited the smallest value. However, the target location with the largest value differed among the animal.

The relative value of Md, a distance of CVP shift during forelimb movement, changed in the range of 0.87–1.59 for Cat 1, 0.70–1.76 for Cat 2, and 0.85–1.55 for Cat 3. It was the minimum for all three cats when the target location was left-front (Figure 7E). In addition, the relative value became large for all cats when targets were located at the right. The relative value of Mt, a time requiring forelimb movement, changed in the range of 0.84–1.20 for Cat 1, 0.67–1.02 for Cat 2, and 0.72–1.06 for Cat 3. Notably, the relative value of Mt was the minimum for all three cats when the target location was at the left-front (Figure 7F). On the other hand, when the target locations were at the right, Mt was rather large in all cats. The relative value of the CVP speed during forelimb movement (M-CVPs) was 1.0–1.36 for Cat 1, 0.85–1.92 for Cat 2, and 1.0–1.74 for Cat 3 (Figure 7G). Like the Md and Mt, while M-CVP was relatively large when the target locations were at the right, no target locations exhibited either the smallest or largest value common to the three cats. Altogether, common to all three animals, the matching score was higher in the left-front target, where the Mt and Md was the minimum, indicating that this target location was the most preferred and the optimal for each cat’s forelimb reaching movement task.

**FIGURE 8:**
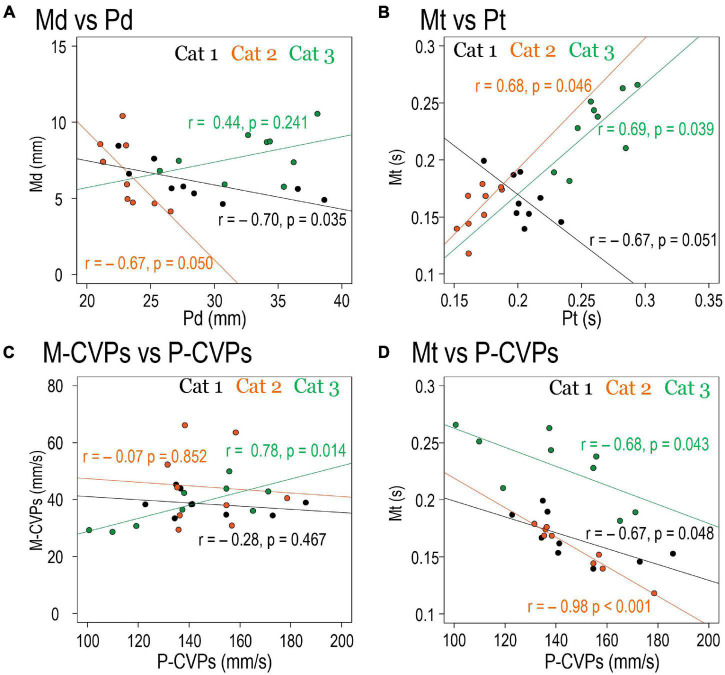
The Relationships between parameters. Each scatters plot shows the correlation between the mean values of each spatiotemporal parameter under nine target conditions of three cats. **(A)** Relationships between Pd and Md. **(B)** Relationships between Pt and Mt. **(C)** Relationships between P-CVPs and M-CVPs. **(D)** Relationships between P-CVPs and Mt.

Since the forelimb reaching movement is triggered following the PPC, it is reasonable to assume that the PPC-related parameters determine the parameters of the forelimb reaching movement. We then examined the relationship between the PPC-related parameters and the movement-related parameters. Results are show in Figure 8. Between spatial parameters, such as Pd and Md, there was significant negative correlations between in Cats 1 (*r* = –0.70, *p* = 0.035) and 2 (*r* = –0.67, *p* = 0.050), but not in Cat 3 (*r* = 0.44, *p* = 0.241; Figure 8A). Between temporal parameters Pt and Mt, Cat 1 had a significant correlation (*r* = –0.67, *p* = 0.051), but Cat 2 (*r* = 0.68, *p* = 0.046) and Cat 3 (*r* = 0.69, *p* = 0.039) exhibited positive correlations (Figure 8B). Between P-CVPs and M-CVPs, parameters of CVP movement speed, no correlation was found in Cats 1 (*r* = –0.28, *p* = 0.467) and Cat 2 (*r* = –0.07, *p* = 0.852). However, Cat 3 showed a positive correlation (*r* = 0.78, *p* = 0.014; Figure 8C). Accordingly, no parameters in the same dimension existed common to all animals between the two postural control processes in the forelimb reaching task. Therefore, as shown in Table 2, we further made correlation analyses between all above parameters (Figure 8D). Eventually, we found it exists that only a combination of P-CVPs and Mt had a strong negative correlation in the 3 animals (*r* = –0.67, *p* = 0.048 for Cat 1, *r* = –0.98, *p* < 0.001 for Cat 2, *r* = –0.68, *p* = 0.043 for Cat 3).

**TABLE 2:** Correlation coefficients between parameters of posture and movement.

	Parameters of movement								
	Md			Mt			M-CVPs		
Cat number	Cat1	Cat2	Cat3	Cat1	Cat2	Cat3	Cat1	Cat2	Cat3
**Parameters of posture**									
Pd	−0.70*	−0.67	0.44	−0.77*	−0.73*	–0.50	–0.56	−0.40	0.86*
Pt	−0.76*	−0.16	0.05	–0.67	0.68*	0.69*	−0.77*	−0.46	–0.50
P-CVPs	–0.50	−0.48	0.22	−0.67*	−0.98*	−0.68*	–0.28	−0.07	0.78*

*p < 0.05.

## Discussion

### Experiment Design and Its Limitation

Many studies reported on the mechanisms of APA. Before the onset of the goal-directed actions, the APA had a role in canceling the postural sway of whole body and local body parts caused by the movements (Massion, 1992). The APA was observed just before the initiation of bipedal gait in humans (Boisset and Do, 2008), the lifting up of either forelimb of cats (Gahéry et al., 1980; Birjukova et al., 1989), and the initiation of the forelimb reaching of the cat (Alstermark and Wessberg, 1985). Schepens and Drew (2003) measured the ground reactive force exerting on the cat extremities together with electromyograms (EMGs) of four legs so that they determined the APA. As a result, the transient load increase that occurred immediately before lifting the paw of the forelimb facilitated the movement of the CVP to the contralateral side, and the authors considered this as the APA in the cat forelimb reaching movement. A transient load increase in the forelimb to reach the target was also observed (Figures 3A, 5) in this study. At the beginning, we focused on the mechanism of generating this inversive postural reaction. However, the magnitude and time of this reaction differed from trial to trial in any cat, even when the target location was changed. Consequently, we detected the apparent inversive reaction in less than 60% of trials.

Lower incidence of this inversive reaction in this study is possibly due to the difference between the experimental design of this study and those of Schepens and Drew (2003). They simultaneously measured the ground reactive force in three directions and EMGs of extremities. They judged the APA by the start of the transient increase in the ground reactive force together with an elevation of EMGs of the triceps brachii muscles. On the other hand, we judged postural change only from the CVP shift calculated from the ground reactive force generated in the vertical direction. Therefore, we cannot negate the possibility of the low detection accuracy of the transient reaction. Another possibility would be the difference in the task setting. Schepens and Drew (2003) used the reaction time task. Canadian cats needed to wait until a go-signal was generated. Such a “ready and go” conditioning might have frequently generated the inversive reactions. On the other hand, we did not use such conditioning. That is, when the cat stabilized one’s posture, the food was thrown into the targeting tube so that the cat spontaneously started the task at one’s timing without waiting. We might test this possibility if we employ the “ready and go” conditioning that they employed. Nonetheless, between trials with and without the transient load increase, we did not detect any difference in changes in ground reactive force other than the transient load increase. Therefore, we defined the postural change from the decrease in the forelimb’s ground reactive force to the moment when the cat lifted its paw as the PPC in this study. In addition, we distinguished it from the APA, which accompanied the transient load increase of the lifting forelimb. We believe, however, that the APA and PPC share common neuronal mechanisms. On the other hand, we should also mention that we did not elucidate on to what extent the cat’s forelimb accurately reached the target. However, considering that the cylinder containing the target was a diameter of 30 mm in which the size was just enough to fit the hand, the analysis accuracy of the reaching movement itself could be sufficiently guaranteed in the trial when the cat reached the target.

Many previous studies, such as Schepens and Drew (2003), used six-axis force transducers. On the other hand, we used one-axis force transducers in this study to elucidate the detailed changes of parameters that depended on the target conditions. To evaluate how the PPC is achieved by means of momentum exerting in each foot will be the subject of our future study. However, it is still meaningful that such a simple recording system is able to evaluate postural changes, especially for human studies on postural control.

### What Parameters Determined the Optimality of the Preceding Postural Control?

As an index of postural control, CVP or center of pressure (COP) has been employed for many studies. For example, in humans, CVP was used to elucidate the postural control when standing at rest (Winter et al., 1996; Gage et al., 2004) and to examine biomechanics at start walking (Jian et al., 1993; Halliday et al., 1998). In addition, Maki et al. (1994) and Quijoux et al. (2020) examined the relationship between CVP agitation and falls in the human standing posture. Benvenuti (2001) proposed several indexes for CVP to elucidate standing posture. Moreover, Kapteyn et al. (1983) pointed out that both the averages of CVP speed and root mean square were valuable indicators of postural stability. Rocchi et al. (2002) used these indexes to quantitatively evaluate the therapeutic effects of deep brain stimulation and L-DOPA on postural sway in Parkinson’s disease patients. In rodents, both movements and distributions of the CVP were also used to examine postural stability (Hutchinson et al., 2007; Funato et al., 2017). In the cat, some studies have focused on the changes in postural synergy during forelimb movements (Gahéry et al., 1980; Birjukova et al., 1989; Schepens and Drew, 2003). However, no studies have quantitatively evaluated the changes in the CVP depending on the target-subject relationship. This study demonstrated that the different target conditions altered the CVP_onset_, CVP_lift_, and CVP_reach_, indicating that the PPC changed the CVP_lift_ position depending on the subject-target relationship using feed-forward mechanisms, or motor programs, based on prediction. In human studies, Day and his collaborators demonstrated that the center of mass (COM) before the onset of the step already had information on the acceleration and speed of the first step (Lyon and Day, 1997; Bancroft and Day, 2016). Yiou et al. (2016) also showed that the pre-step activity altered depending on the final position and distance of the foot. Together with our findings, these results are consistent with the description by Mille et al. (2012) that “the anticipatory nature of the postural step coordination appears to involve a role for motor prediction.”

We observed that Md, the difference between CVP_reach_ and CVP_lift_, altered depending on the target’s location. For example, while Md was the smallest when the target was in the left-front (Figure 5A), it was large if targets were far to the right (Figure 5B). In the former condition, the distributions of CVP_lift_ and CVP_reach_ overlapped in more than 80% of trials. Accordingly, as mentioned earlier, the cat possibly predicted the posture of the end of the task before starting the action, and Md can be the “prediction difference.” When Md was large, cats were required to simultaneously perform both goal-directed forelimb reaching movement and accompanying postural control. Considering that both of these processes require visuomotor processing of the higher-order brain function, the reaching task under the latter condition requires higher cortical activity than the former. Therefore, we corroborate that the change in Md associated with the target location difference can be a candidate parameter that may contribute to anticipating the visuomotor processing capability.

When the target was left-front, not only Md but Mt (the time required for reaching movement) was the minimum in all cats. Suppose this result applies to Fitts’ law (speed-accuracy trade-off, Fitts, 1954), the smaller the Mt, the higher the performance of the reaching movement, suggesting that the goal-directed action became to be more optimized. Therefore, the fact that Mt became the minimum together with Md at the left-front target location indicates that this target location corresponded to the optimal target-subject relationship. Consequently, we further postulate that the functional role of the PPC can be to ensure the stability of the posture during the entire period of the goal-directed movement by adjusting Md and Mt so that it can optimize the action. This postulation fits well with the consideration by Do et al. (1992) and Boisset and Do (2008), who described how “postural control is programmed in relation not only to the focal movement parameters *per se* but more generally to task movement parameters.”

Then, which of the PPC parameters determined postural control during the forelimb reaching movement? We examined the correlation between parameters of the PPC and those during reaching movement in three cats (Table 2). Only the Mt and P-CVPs combination showed a negative correlation in the three cats (Figure 8D). This finding indicates that reaching movement required time to perform under the target condition where the speed of the CVP shift during PPC was slow. Moreover, Birjukova et al. (1989) found, in the cat forelimb reaching task, that speed of COP shift on the base-of-support during postural control before the onset of forelimb movement reduced after removal of the sensorimotor cortex. Their findings, together with the present results, suggest that P-CVPs can be not only the determinant of the Mt but also the indicator reflecting the cortical function of integrating target-subject interaction.

Furthermore, these animal studies’ findings are significant to understanding pathophysiological mechanisms of postural disturbances in neurological disorders. Namely, patients with Parkinson’s disease and cerebrovascular disorders showed the reduced speed in COP shift that occurred before the onset of arm-hand reaching movements in upright standing posture (Porter et al., 2016; Portnoy et al., 2017). More specifically, damages in APA during gait initiation in stroke patients is characterized by atypical patterns (Rajachandrakumar et al., 2017), longer duration (Hesse et al., 1997; Sousa et al., 2015), lower velocity (Gama et al., 2019), and lower amplitude (Melzer et al., 2010; Sharma et al., 2015). Accordingly, there is a need to elucidate whether the above parameters can be beneficial to judge the stability of postural control of patients with neurological disorders and can be the predicting maker of falling. For this, studies combined with biological research, biomechanics, and control theory should be required (Johansson et al., 2009; Pettersson et al., 2012; Kaminishi et al., 2020, 2021).

### Commonalities and Differences in the Preceding Postural Control in Each Animal

There is a need to mention the commonalities and differences in each cat. To identify the postural control mechanism common to all cats, we examined how PPC altered by changing the target conditions (nine conditions). As a result, we found that the speed of the PPC had an inverse correlation with Mt in all cats, as shown in Figure 8D. On the other hand, there was a positive correlation between Mt and Pt in Cats 2 and 3, but Cat 1 exhibited a negative correlation between the two parameters (Figure 8B). However, there is a possibility that the obtained correlation was spurious since we only used nine conditions (nine measurement points) for statistical analyses. Therefore, we employed regression analysis to see if the correlation was significant so that we overcame the drawback of having few measurement points.

The following commonalities existed in each cat. Regarding CVP, there was a substantial overlap in the distribution of CVP_lift_ and CVP_reach_. Such an overlap, a matching score, was much higher when the target was at the left-front position. However, as the target moved to the right or backward, the distribution of CVP_lift_ and CVP_reach_ also changed, suggesting that each cat performed PPC based on the spatial relationship between oneself and the target. Therefore, the cortical visuomotor process may contribute to both the forelimb reaching movement and the PPC. Furthermore, each cat exhibited a negative correlation between the postural CVP speed and the forelimb movement time (Mt), indicating that the PPC coded the temporal parameter of the purposeful movement in addition to the posture at the end of the movement. Based on these considerations, we postulate that the common motor program can produce both the PPC and goal-directed movements based on cortical visuomotor processes.

On the other hand, the following were not common to each cat. First, the inversive response before the onset of PPC was 50–60% for Cat 1 and 2 but 26% for Cat 3. Second, the CVP_onset_ of Cat 1 and 2 moved depending on the target conditions, but not the case in Cat 3. These differences may be due to the tasks used in this study. As mentioned earlier, if we employed the reaction time task as used by Schepens and Drew (2003), the inversive reaction might occur in all cats. Moreover, if the inverse reaction further requires the cat’s attention against the target, CVP_onset_ in each animal may also alter depending on the change in the target position. An alternative interpretation would be that the inversive reaction depends on the age of the subject. When we made this study, Cats 1, 2, and 3 were 6, 5, and 1 year old, respectively. As described in the “Introduction” section, the younger subject did not often exhibit inversive reactions when starting the step (Hyodo et al., 2012). Therefore, we cannot disregard the possibility that the incidence of the inversive response is lower in younger cats than in elder cats. In addition, while there was a positive correlation between Mt and Pt in Cats 2 and 3, Cat 1 exhibited a negative correlation between the two parameters (Figure 8B). This indicates that the PPC may have coded even the time required for forelimb reaching with different strategies in each cat.

### Possible Neuronal Mechanisms of the Preceding Postural Control

Because PPC precedes goal-directed movements, descending pathways other than the lateral corticospinal tract may be involved in the PPC. For example, in the cat, Yakovenko and Drew (2009) demonstrated that pyramidal neurons in the motor-related cortical areas exhibited higher activity before starting the forelimb reaching movement. Mori et al. (2003) observed that injection of muscimol, a GABAergic agonist, into monkeys’ supplementary motor area (SMA) made it difficult to maintain posture during bipedal locomotion on the treadmill. Moreover, a muscimol injection into the dorsal premotor area (PMd) disturbed sensory-guided locomotion. In a clinical study, Jacobs et al. (2009a) reported that transcranial magnetic stimulation (TMS) to SMA altered APA in healthy individuals and patients with Parkinson’s disease. Therefore, the motor-related cortical areas such as SMA and PM, which generate motor programs, may play a crucial role in the PPC and APA. Because abundant fibers exist projecting from these motor areas to the pontomedullary reticular formation (Keizer and Kuypers, 1989; Matsuyama and Drew, 1997), the PPC or APA is possibly mediated by the cortico-reticular projection and the reticulospinal tract. In addition, the motor-related cortical areas may send a program of forelimb reaching to the primary motor cortex (M1), which evokes goal-directed movements via activation of the lateral corticospinal tract. Evidence showed that the damages in the cerebellum (Timmann and Horak, 2001; Bruttini et al., 2015) and the basal ganglia (Jacobs et al., 2009b; Ng et al., 2013) also disturbed the APA, indicating that the motor loop between the motor-related cortical areas with the cerebellum and basal ganglia are involved in both the motor programming and execution of the PPC.

In this study, the cat PPC predicted and provided the posture at the end of forelimb reaching movement even under the condition of different target locations. This result suggests that the cat recognized the relationship between the target and oneself in space and reflected this in the motor program of both the PPC and forelimb reaching movement. The cognitive information required to generate this motor program is produced in the parietal association area. This area is considered to integrate sensory information necessary for the maintenance of upright posture (Bisdorff et al., 1996; Peterka, 2002; Barbieri et al., 2008) and to produce spatial cognitive information and internal models of the self-body (Andersen, 1997; Andersen and Buneo, 2002). This cognitive information is possibly sent to the motor-related areas such as SMA via the abundant projection from this region (Luppino et al., 1993). Furthermore, we recently observed that microinjection of muscimol into the parietal cortex of the cat altered both the forelimb reaching movement and the PPC (Takahashi et al., 2019a,b). In other words, the parietal association area may play a significant role in postural control, and dysfunction of this area may disturb postural control, resulting in falling.

## Conclusion

We demonstrated that the postural control that preceded the forelimb reaching movement of the cat predicted the posture at the end of the movement and provided it before starting the movement. We also showed that optimization of the subject-target relationship achieved the preceding postural control that induced high-performance forelimb reaching movement. We conclude, therefore, that the motor program generated based on the cognitive visuomotor processes may accomplish the PPC in addition to the goal-directed movement.

## Author Note

This manuscript was also published in the Ph.D. thesis of author MT.

## Data Availability Statement

The raw data supporting the conclusions of this article will be made available by the authors, without undue reservation.

## Ethics Statement

The animal study was reviewed and approved by Animal Studies Committee of Asahikawa Medical University.

## Author Contributions

MT and KT conceived and designed the experiment. MT and TN acquired and analyzed the data. MT, TN, and KT drafted and revised the manuscript and gave final approval to the submitted version.

## Conflict of Interest

The authors declare that the research was conducted in the absence of any commercial or financial relationships that could be construed as a potential conflict of interest.

## Publisher’s Note

All claims expressed in this article are solely those of the authors and do not necessarily represent those of their affiliated organizations, or those of the publisher, the editors and the reviewers. Any product that may be evaluated in this article, or claim that may be made by its manufacturer, is not guaranteed or endorsed by the publisher.

## Footnotes

1
https://www.R-project.org/
